# Multiple scattering effects on intercept, size, polydispersity index, and intensity for parallel (VV) and perpendicular (VH) polarization detection in photon correlation spectroscopy

**DOI:** 10.1038/s41598-020-78872-4

**Published:** 2020-12-10

**Authors:** Ragy Ragheb, Ulf Nobbmann

**Affiliations:** Malvern Panalytical, 117 Flanders Road, Westborough, MA 01581 USA

**Keywords:** Materials science, Techniques and instrumentation, Characterization and analytical techniques

## Abstract

Dynamic light scattering (DLS) is well established for rapid size, polydispersity, and size distribution determination of colloidal samples. While there are limitations in size range, resolution, and concentration, the technique has found ubiquitous applications from molecules to particles. With the ease of use of today’s commercial DLS instrumentation comes an inherent danger of misinterpretation or misapplication at the borderlines of suitability. In this paper, we show how comparison of different polarization components can help ascertain the presence of unwanted multiple scattering, which can lead to false conclusions about a sample’s mean size and polydispersity. We find that the contribution of multiple scattering events effectively reduces both the measured scattering intensity and the apparent size from the autocorrelation function. The intercept of the correlation function may serve as an indicator of relative strength of single to multiple scattering. Furthermore, the abundance of single scattering events at measurement positions close to the cell wall results in an apparent increase in uniformity yielding a lower polydispersity index which is more representative of the physical system.

## Introduction

In dynamic light scattering (DLS), the scattered light from a dispersion containing diffusing particles is correlated with itself. This technique is also known as photon correlation spectroscopy. Typically, the intensity-intensity autocorrelation function, G(τ), then shows a decay rate that is directly related to the Brownian movement of the scattering objects^1–3^. Equation 1 (below) expresses the intensity-intensity autocorrelation function for the ideal experimental scenario of identical particles with diffusion coefficient, D, using the Siegert relationship^4^.

1 $$ G\left( \tau \right) = 1 + \beta \left\{ {{\text{exp}}\left( { - Dq^{2} \tau } \right)} \right\}^{2} $$

Here, the exponential decay as a function of delay time, τ, represents the loss of coherence due to Brownian motion with diffusion coefficient, D, and wave vector, q. The signal-to-noise factor, β, expresses the overall optical detection efficiency, also known as the coherence factor or Y-intercept. In a generalized form of the above equation, the exponential decay, in {} brackets, can be replaced with an integration of D and q, from different particles and/or different scattering vectors, respectively. The most common interpretation of the result is the assumption of single scattering. In that case, all detected photons arriving at the detector are assumed to have encountered and scattered off at maximum one scattering object, with the same defined scattering vector. The scattering vector is given by the equation below where *n* is the solvent refractive index, λ is the wavelength of the laser, and θ the scattering angle within the sample of the experimental setup.

2 $$ q = \frac{4\pi n}{\lambda }{\text{sin}}\left( {\frac{\theta }{2}} \right) $$

The decay rate of each single-scattering exponential decay function leads to the corresponding translational diffusion coefficient, *D*, given by the thermal energy divided by the viscous drag of a particle. This is also known as the Stokes–Einstein equation^1–3^

3 $$ D = \frac{{k_{B} T}}{{6\pi \eta R_{H} }} $$

where η is the dispersant viscosity, *T* is the absolute temperature, *k*_*B*_ is the Boltzmann constant, and *R*_*H*_ the hydrodynamic radius. The Cumulant method is commonly used to obtain an average size in DLS as described by ISO 13321. Experimental data are essentially “force-fit” to the autocorrelation function in Eq. 1. The logarithmic plot of [G(τ)-1] is fit to a polynomial expression where the first and second order fitting terms produce the average size (z-average diameter) and the polydispersity index (PDI) per Eq. 4.

4 $$ \ln \left[ {G\left( \tau \right) - 1} \right] = \ln \beta - 2 a_{1} \tau + 2 a_{2} \tau^{2} + \cdots $$

With

5 $$z_{ave} = \frac{k_{B} Tq^{2}}{{3 \pi a_{1} }} \, \,\,\,\text{and}\,\,\,\,PDI = \frac{2 a_{2}}{a_{1}^{2}}$$

This approximation is particularly valid at short delay times τ and for similar particles. Furthermore, the z-average is an intensity weighted mean of the particle distribution and the PDI exhibits the variance in size, indicating the normalized width of a Gaussian distribution.

The concept of single scattering is at the core of practically all commercial particle sizing instruments that are based on dynamic light scattering. As the concentration of scattering centers in a sample gets higher, we then start to encounter a phenomenon where photons face an increased likelihood of scattering off neighboring particles prior to reaching the detector. In other words, the path of a typical photon may involve multiple scattering centers, thus termed multiple scattering. Initial theoretical and experimental studies of this situation looked at the onset and influence of double and multiple scattering contributions^5,6^. Sorensen et al. (1976) demonstrate that the lowest order contribution to depolarized scattering in non-interacting samples comes from double scattering events. They show that the correlation from these double scattering events is independent of scattering angle and approximates the single-scattering correlation function for a scattering angle of 180°. Our experimental scattering angle of 173° in this study is very close to that 180°, and thus the depolarized double-scattering should lead to a very similar z-average. In Sorensen et al. 1978, the depolarization ratio of depolarized to polarized intensity is related to the average number of scattering events. Sorensen et al. studied a distinctly lower number of scattering events. This paper extends the study to more scattering events but cannot directly compare to Sorensen’s work due to differences in the experimental setup.

DLS analyzes the intensity fluctuations in the scattered light from objects moving in the light path. Single scattering probes the movement of individual particles. Multiple scattering reflects many particles in a typical light path with increasing sensitivity to each other and causing faster fluctuations. When multiply scattered light is interpreted as single scattering, this leads to more rapid decay in the correlation function, and thus a smaller apparent size than they really are. Further observable effects of multiple scattering from higher particle concentrations include a decrease in the Y-intercept of the correlogram, a decrease in apparent average size, and an increase in polydispersity.

While there are advanced experimental cross-correlation solutions to overcome the influence of multiple scattering^7,8^, the most commonly applied experimental steps to limit or suppress the influence of multiple scattering include.

**Figure 1:**
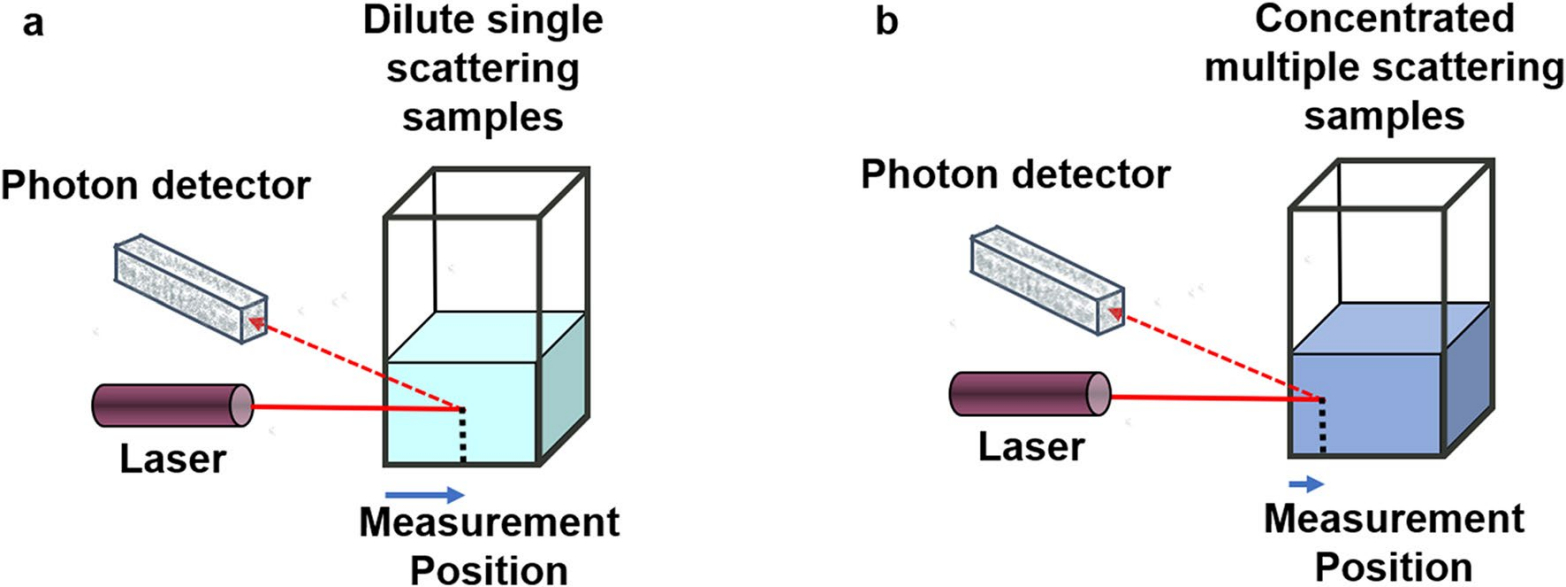
Schematic detailing the Non-Invasive Back Scatter (NIBS) optical configuration with the laser and the photon detector on the same horizontal plane perpendicular to the vertical direction of the cuvette and the automated measurement positioning process used to accommodate high concentration dynamic light scattering measurements with the Zetasizer for dilute samples (**a**) detection at the cuvette center, for turbid sample (**b**) close to the cuvette wall.

- diluting down the sample (Fig. 1a) or
- shortening the path length between the scattering volume center and the cuvette surface^9^ (Fig. 1b).

**Figure 2:**
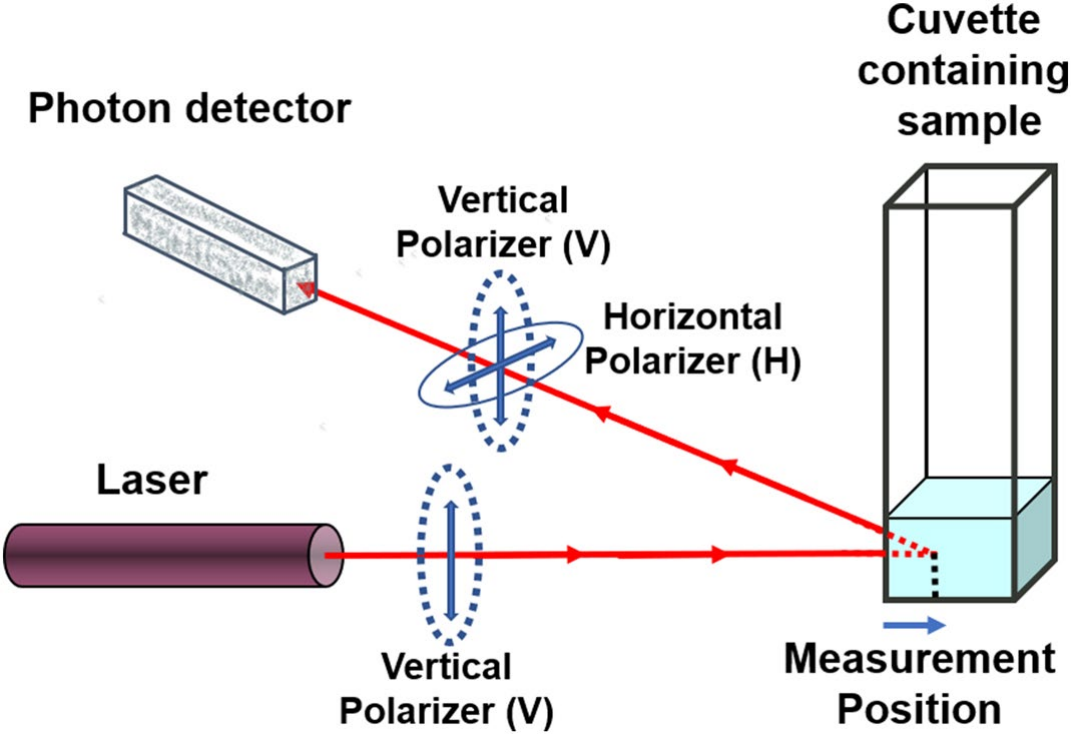
Diagram showing the light from a vertically polarized laser (where the laser is on the same horizontal plane as the photon detector perpendicular to the vertical direction of the cuvette) going through the sample where the measurement position in the cell is noted, followed by the inclusion of the vertical and horizontal polarizers between the cell and photon detector. This is further denoted as VV and VH to reflect the respective input and output polarization.

A typical Dynamic Light Scattering setup incorporates a vertically polarized laser beam illuminating the sample. It is important to note that the laser and the photon detector sit on the same horizontal plane. Light scattered from this sample is detected at a defined scattering angle, and the intensity fluctuations are analyzed. When a polarizer is inserted into the path between the sample and the detector, the observed light scattering intensity will vary depending on the polarizer orientation with respect to the horizontal scattering plane. VV and VH are common orientations where VV refers to the vertical input polarization of the laser and vertical polarization at the detector and VH refers to vertical input polarization of the laser and horizontal polarization at the detector. The VV arrangement is often referred to as parallel polarization detection and VH is called perpendicular polarization detection. The amount of detected light is dependent on both the scattering angle and the polarizer orientation before/at the detector. The Zetasizer Ultra incorporates both VV and VH polarizer arrangements (Fig. 2).

As a “real world” sample, we chose milk, the staple in the dairy industry. The distribution of whey proteins, casein micelles, and milk fat emulsion droplets in water have been analyzed by DLS in the past^10–13^. Mootse et al. discovered they had to dilute their samples to bypass any multiple scattering when measuring raw milk^11^. Beliciu et al. furthermore, explored the choice of different diluting solvents to avoid multiple scattering of milk samples^10^. Urban et al. and Sinn et al. both used 3D cross-correlation to characterize turbid suspensions and discovered they could measure samples at stock concentrations without issue^12,13^. In this paper, we explore how multiple scattering of stock commercial whole milk as an analyte with both high and low concentrations at different measurement positions affect the scattered light detected through a vertical and horizontal polarizer.

## Experimental

Simply Nature Organic Vitamin D DHA OMEGA-3 Whole Milk was measured on a Malvern Panalytical Zetasizer Ultra (Malvern Panalytical, Westborough MA) at a detection angle of 173°. The scattering angle of 173° is in air. The actual angle will depend on the RI of the dispersant. The uncertainty on this, based on the specification of the single mode optical fiber, is ± 0.4°. All measurements in this study were taken at a temperature of 25 ± 0.1 °C, 5 repeat measurements at measurement positions every 0.25 mm from 0.89 to 4.64 mm in a disposable sizing cuvette (part number: DTS0012). Cuvettes were removed from a carefully covered box and used as-is. All data points shown in graphs below include error bars from the 5 repetitions but due to the minimal standard deviation, they might not be visible. The sample was further diluted in distilled water with a negligible derived mean count rate average of 68 ± 8 kcps and compared to the stock. Furthermore, each series of positions was measured without a polarizer, with a vertical polarizer and with a horizontal polarizer. The polarizers used on the Zetasizer are a dichroic film with an extinction ratio of > 5000:1 for wavelengths 530–690 nm. The instrument has a built-in polarizer selection wheel and uses a 10 mW He–Ne laser operating at a wavelength of 633 nm. The general-purpose method algorithm in the software (ZS Xplorer, version 1.2.0.91) was used to analyze the correlation functions. The DLS technique measures the diffusion coefficients of molecules or particles undergoing Brownian motion. The Stokes–Einstein equation is used to calculate the hydrodynamic size from the diffusion coefficient. In DLS, the signal strength is the scattering intensity. It is measured in photons per second, often also referred to in unit of kilocounts (of photons) per second (kcps). The actual average number of photons per second arriving at the detector is the mean count rate or scattering intensity. The instrument has a range of different attenuators to regulate the amount of light going to the detector. The automatic mode in the software will select an attenuation level to achieve a mean count rate in the optimal detection range of 200–500 kcps for this particular instrument’s configuration and to insure proper statistical analysis. The Derived Count Rate (DCR) takes the actual attenuation level selection into account and represents the theoretical count rate at 100% laser power. The laser beam is attenuated using a ND (neutral density) filter with known attention values so the uncertainty of the DCR is the same as the uncertainty of the measured count rate, which is assessed by recording in replicate. This DCR is a useful metric to compare the signal strength from different samples. For the same sample material, a higher DCR usually indicates a higher concentration, larger particles, or a combination of both higher concentration and larger particles.

## Results and discussion

**Figure 3:**
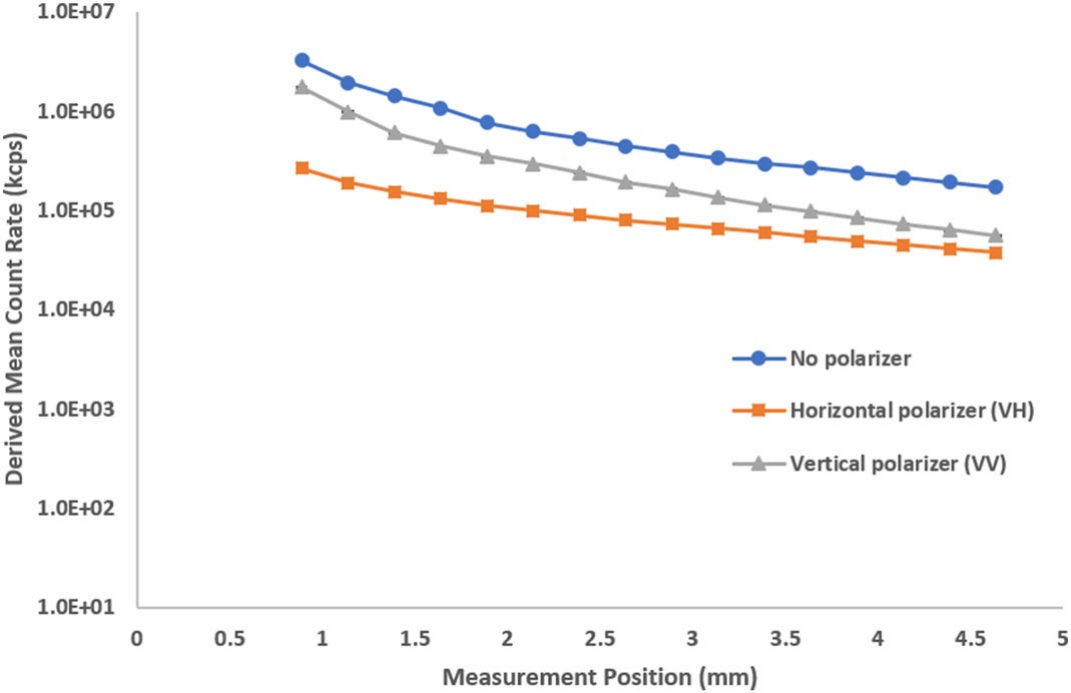
Derived count rate as a function of measurement position at different polarizer configurations for stock milk. The influence of multiple scattering increases further into the sample, away from the cuvette wall, leading to an apparent decrease in scattering intensity.

**Figure 4:**
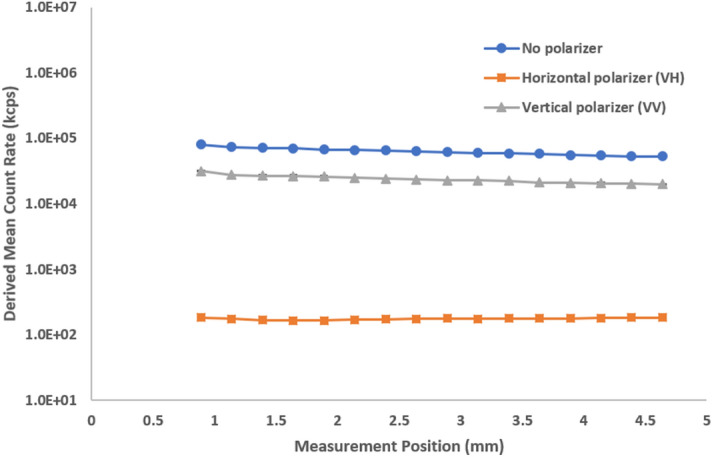
Derived count rate as a function of measurement position at different polarizer configurations for 1000-fold diluted milk. The dilution exhibits more single scattering with minimal change in count rate over position and no cross-polarized signal (no signal in the horizontal plane).

The first plot shows the derived mean count rate (in kcps) as a function of measurement position (Fig. 3). We analyzed the sample without a polarizer, with the horizontal polarizer, and with the vertical polarizer. We observe a general decrease in the DCR for all three traces because multiple scattering events in a typical path increase as the measurements position goes further into the cuvette. The milk, when diluted 1000 × fold, (Fig. 4) exhibited single scattering with significantly less decrease as a function of measurement position and minimal cross-polarized signal (low signal in the horizontal plane). With multiple scattering, not all vertically polarized input light will yield vertically polarized light at the end of the scattering path. The polarization becomes increasingly random, resulting in less vertical polarization and increasing contributions of horizontal polarization, outside the traditional scattering plane. Completely random polarization would theoretically be comprised of half vertical and half horizontal contributions by intensity. The more multiple scattering present in a sample, the more random the polarization contribution of all scattering events. Perpendicularly polarized scattered light (horizontal, VH) will primarily exhibit multiple scattering while parallelly polarized (VV) will primarily exhibit single scattering events. A similar comparison was made with 300 nm latex standards before and after a 100-fold dilution (Supplementary Figs. S6 and S7). Stock 20 nm latex standards exhibit completely single scattering (Supplementary Fig. S2). These samples serve as a model system for the presence or absence of multiple scattering when compared to a more complex “real world” sample such as milk (Supplementary Fig. S1). When we compare the results of no polarization to VH and VV we note that:

- A polarizer will reduce the detected intensity.
- Close to the cuvette wall, the parallel polarization (VV) is stronger than the perpendicular (VH) detection.
- In highly multiple scattering samples, away from the cuvette wall, both polarization components become equal.

We clearly observe these effects in Fig. 3. Through all measurement positions the relationship “No polarizer > VV > VH” holds for the scattering intensity.

**Figure 5:**
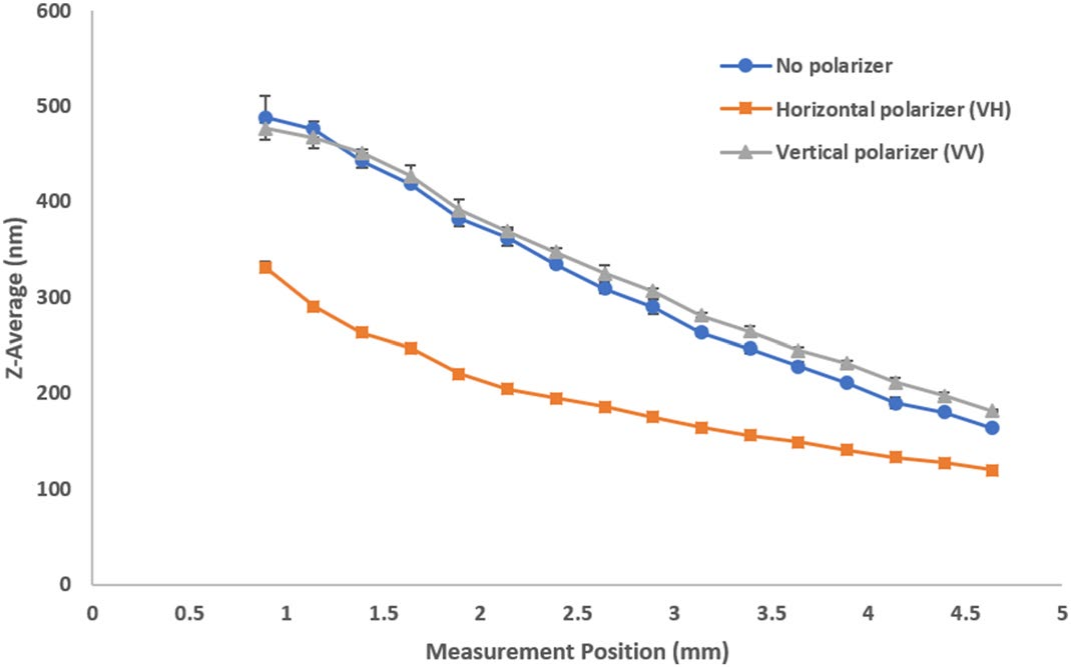
Size (z-average diameter) as a function of measurement position shows the effects of multiple scattering with different polarizer configurations for stock milk. The significance and influence of multiple scattering increases further into the sample, away from the cuvette wall.

**Figure 6:**
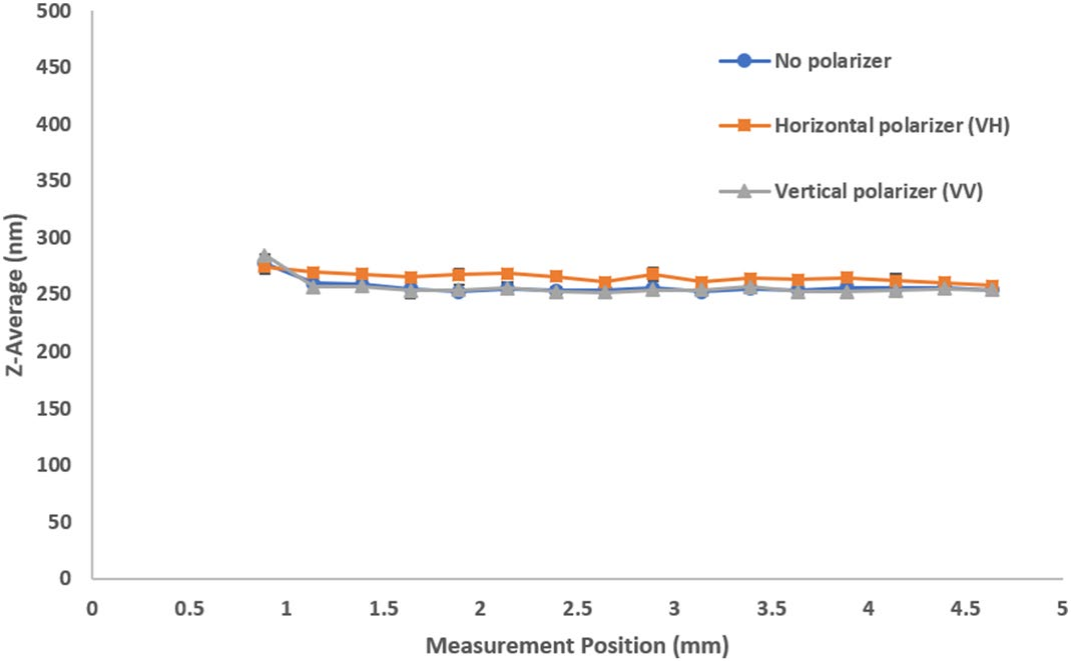
Size (z-average diameter) as a function of measurement position shows the absence of multiple scattering with different polarizer configurations for 1000-fold diluted milk. The sample shows single scattering with consistent sizes over position regardless of polarizer configuration.

While there are theories on highly multiple scattering^14,15^, this paper concentrates on the commonly used z-average which is the overall average or cumulant size from a single exponential fit to the correlation function, as previously defined in Eqs. 4 and 5. Plotting the mean size versus measurement position and different polarization detections for stock milk in Fig. 5 shows multiple scattering causing a decrease in size as the measurement position approaches the cuvette center. This reflects the presence and relatively higher contribution from multiply scattered photon paths to the correlation function. The parallel polarization component (VV) shows the relative abundance of single scattering events and a general increase in apparent size while the perpendicular polarization component (VH) conversely shows more multiple scattering and smaller apparent sizes regardless of measurement position. Diluted milk shows single scattering with a consistent size over measurement positions (Fig. 6). It is important to note that the shortest measurement positions reflect the least amount of multiple scattering. However, the difference in size between the stock and diluted milk at these short measurement positions can potentially be attributed to the effects of diluting a complex fluid like milk with water. This effect is absent when diluting a simple 300 nm latex sample where sizes at shorter measurement positions are consistent between stock and diluted standards. Please refer to the corresponding results for model latex standards (Supplementary Figs. S3, S8, and S9 for 20 nm, 300 nm, and diluted 300 nm latex standards respectively).

When we compare the size results of no polarization to VH and VV we note that:

- A parallel polarizer (VV) will increase the selection of single scattering over no polarizer.
- A perpendicular polarizer (VH) will increase the selection of multiple scattering.
- In opaque samples, the contribution of multiple scattering increases away from the wall, regardless of polarizer selection.

We clearly observe these effects in Fig. 5. For this opaque sample the general relationship “VV > No polarizer > VH” holds for the apparent size. Not shown in Fig. 5, is that this relationship breaks down directly at (or very close to) the cuvette wall: Here, the contribution of VV flare leads to mixing of static flare from the cuvette wall with the scattered signal, leading to an artificial contribution to the correlation function that corresponds to half the size.

**Figure 7:**
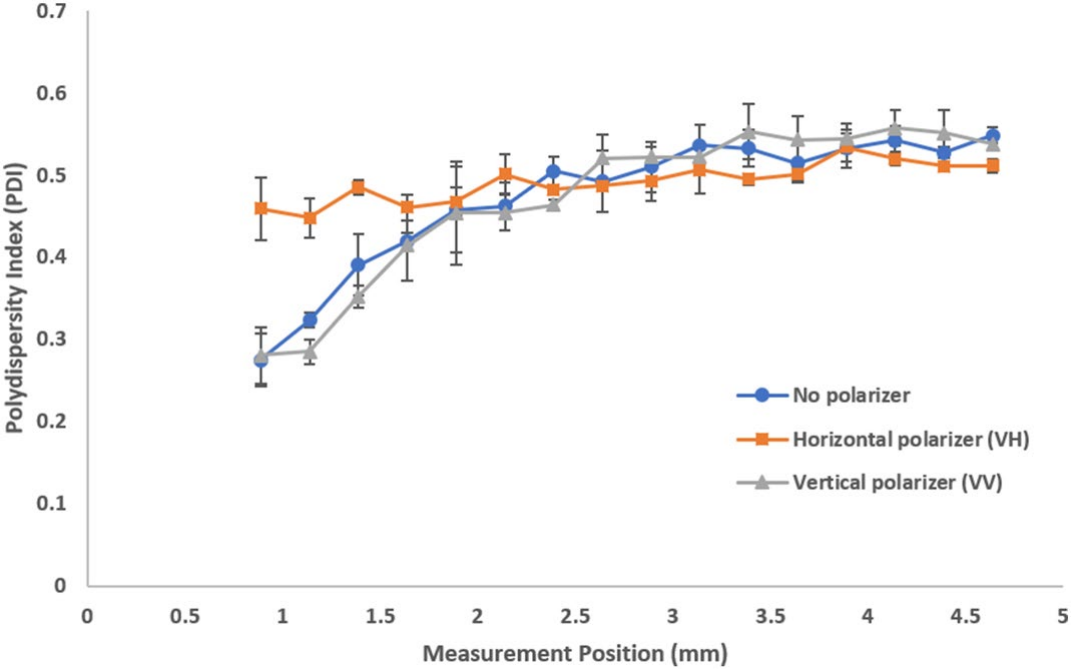
Polydispersity index (PDI) as a function of measurement position shows the effects of multiple scattering with different polarizer configurations for stock milk. The effects of multiple scattering are most evident at positions closer to the cell wall.

**Figure 8:**
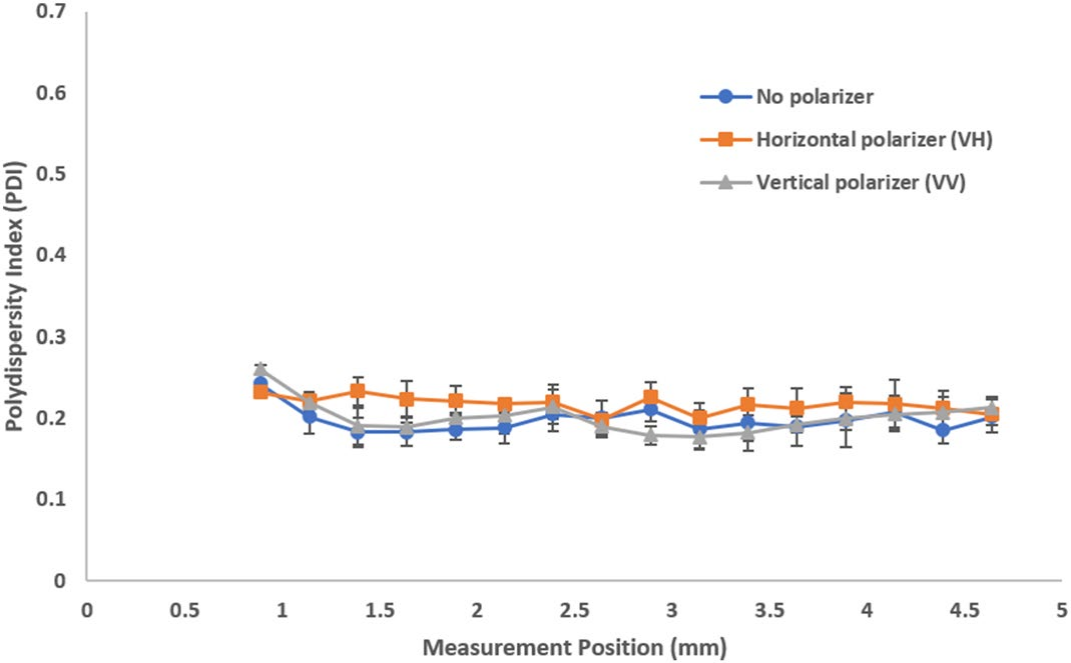
Polydispersity index (PDI) as a function of measurement position shows the absence of multiple scattering with different polarizer configurations for 1000-fold diluted milk. Single scattering shows consistent and lower PDIs regardless of polarizer configuration.

We further look at the effects that polarizers have on the observed polydispersity in stock milk (Fig. 7). The VH configuration exhibits higher PDIs closer to the cell wall than the VV configuration or absence of any polarizer. This suggests that the relatively higher contribution of the multiply scattered photons broadens the size distribution. When the VV configuration or no polarizer is selected, the relative abundance of single scattering contributions closer to the cell wall results in an apparent increase in uniformity, yielding a lower PDI. As the measurement position moves further into the cuvette, the different polarizer configurations become indistinguishable with the overwhelming influence of multiple scattering. The 1000-fold diluted milk exhibits single scattering with consistent and lower PDIs regardless of both measurement position and polarizer configuration (Fig. 8). The effects on PDI were further confirmed with model latex standards (Supplementary Figs. S4, S10, and S11 for 20 nm, 300 nm, and diluted 300 nm latex standards respectively).

**Figure 9:**
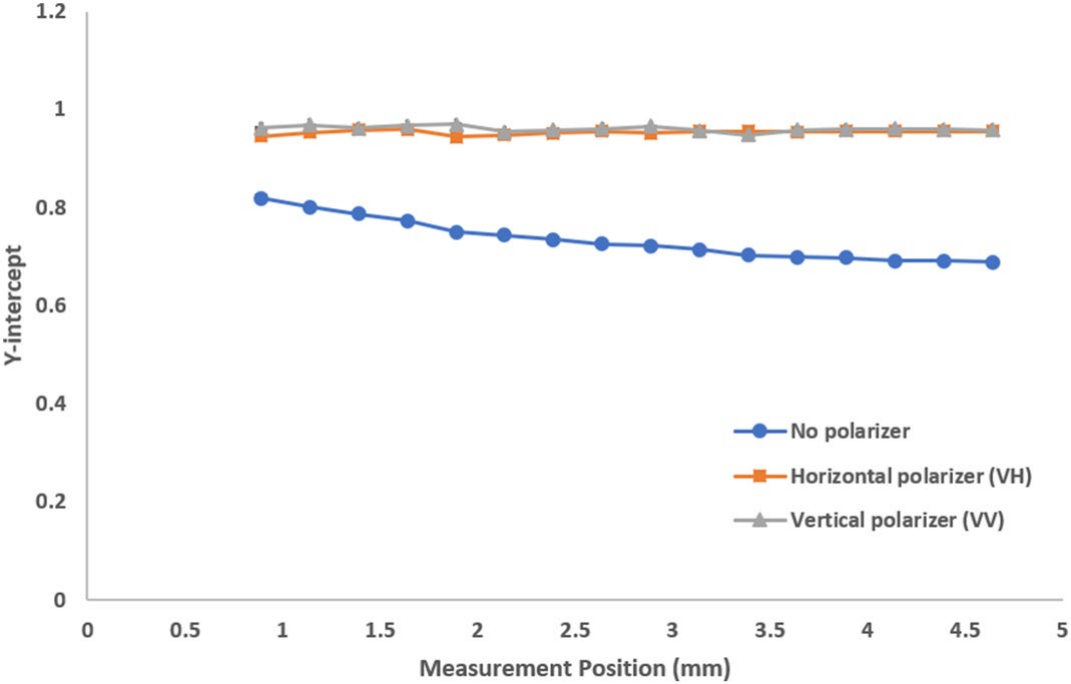
Y-intercept as a function of measurement position for stock milk showing increased presence of multiple scattering away from the cuvette wall. For reasonable scattering intensity, any polarizer detection will lead to a near-perfect intercept approaching 1.

**Figure 10:**
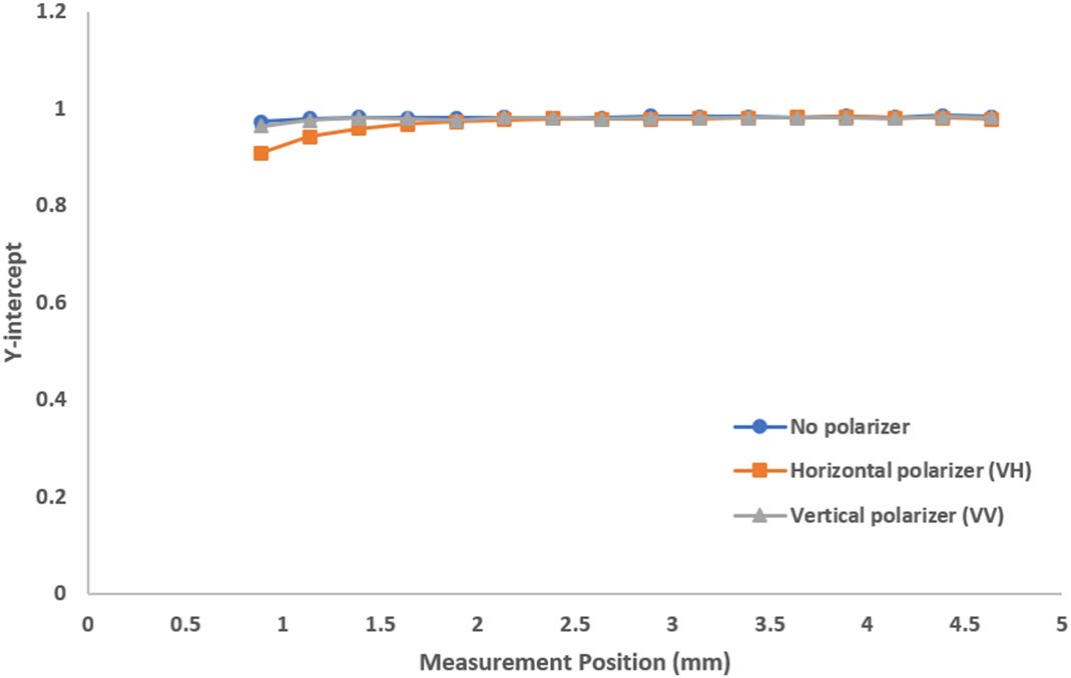
Y-intercept as a function of measurement position for 1000-fold diluted milk showing reasonable scattering intensity, any polarizer detection will lead to a near-perfect intercept approaching 1.

Finally, we consider how the presence or absence of multiple scattering can affect the intercept of the correlation function. This parameter is often an indicator of data quality^16^. A perfectly normalized correlation function has an intercept of 1.0, however this is often not achievable due to noise contributions and optical aberrations. By selecting a dedicated polarization component, we reduce the influence of noise generators and achieve nearly perfect intercepts, provided we have enough scattering signal. As shown in Fig. 9, we see that the Y-intercept is nearly ideal for both horizontal and vertical polarizers. The intercept in an opaque sample is reduced in the absence of the polarizer. We expect light from all polarization planes would contribute equally for a perfectly multiple scattering sample. The unpolarized correlation function can then be considered as a contribution of two independent (and orthogonal) polarization components. Thus, the best achievable intercept for complete multiple scattering is 0.5. As we detect further into the cuvette, the relative contribution of multiple scattering to the ‘No polarizer’ result increases, and its intercept gets closer to the theoretical limit of 0.5. Diluted milk further demonstrates single scattering with a near ideal intercept regardless of measurement position and polarizer configuration (Fig. 10). A similar effect on intercept was demonstrated with 300 nm latex standards before and after a 100-fold dilution (Supplementary Figs. S12 and S13). Stock 20 nm latex standards exhibit completely scattering with consistent Y-intercepts near 1 regardless of measurement position (Supplementary Fig. S5).

The intercept results of no polarization, VH and VV indicate that:

- Any polarizer (VV, VH) will increase the intercept.
- Detection without any polarizer will show a lower intercept due to multiple scattering.
- In opaque samples, the contribution of multiple scattering increases away from the wall.

For this opaque sample the general relationship “0.5 < No polarizer < VH = VV” holds for the intercept of the (symmetrically normalized) intensity-intensity autocorrelation function.

## Conclusion

Comparing opaque and diluted dispersions, we investigated the influence of multiple scattering in a typical DLS measurement. We demonstrate that inserting a parallel polarizer before the detector, the VV configuration, enhances the relative contribution of single scattering. Conversely, by inserting a perpendicular polarizer before the detector, the VH configuration, reduces the relative contribution of single scattering. When no specific polarization is selected, we found that the presence of multiple scattering decreases the correlation function intercept, the scattering intensity, and apparent size and increases the polydispersity in stock milk. Diluted milk shows consistent values for all these parameters regardless of the measurement position. Multiple scattering in undiluted milk could lead to drastic misinterpretation of the results. There was about a 2.5-fold difference in average size between undiluted and diluted milk based on measurement position. Looking at contributions from both VV and VH detection versus no polarizer detection may help to ascertain the presence or absence of multiple scattering, and thus avoid incorrect interpretation of dynamic light scattering results.

## Supplementary information

Supplementary Information.
