# Supplementary material for: Multiple scattering effects on intercept, size, polydispersity index, and intensity for parallel (VV) and perpendicular (VH) polarization detection in photon correlation spectroscopy
Source: Sci Rep. 2020 Dec 10;10:21768. doi: 10.1038/s41598-020-78872-4 (PMC7729959; doi:10.1038/s41598-020-78872-4)
Supplement: Supplementary file 1 — Supplementary Information. [file 41598_2020_78872_MOESM1_ESM.docx]

**Electronic Supporting Materials**

**Authors** Ragy Ragheb^1^ and Ulf Nobbmann^1^

**Title** Multiple scattering effects on intercept, size, polydispersity index, and intensity for parallel (VV) and perpendicular (VH) polarization detection in photon correlation spectroscopy

^1^Malvern Panalytical, 117 Flanders Road, Westborough, MA 01581 USA

[ragy.ragheb@malvernpanalytical.com](mailto:ragy.ragheb@malvernpanalytical.com)

We further demonstrate the presence and absence of multiple scattering with a set of Polystyrene polymer particle standards in water (Thermofisher Scientific). We used a 20nm latex standard (LTX 3020A, 1% solids), 300nm undiluted latex standard (LTX 3300A, 1% solids), and 300nm standard diluted 100-fold in water as an analyte with both high and low concentrations (Figure S1). The stock 20nm and diluted 300nm were used to demonstrate single scattering. Furthermore, the stock 300nm standard was selected to emulate the particle size and concentration and subsequent multiple scattering found in milk. We demonstrate the effects of concentration and measurement positions on scattered light detected through a vertical and horizontal polarizer as described in the main manuscript.


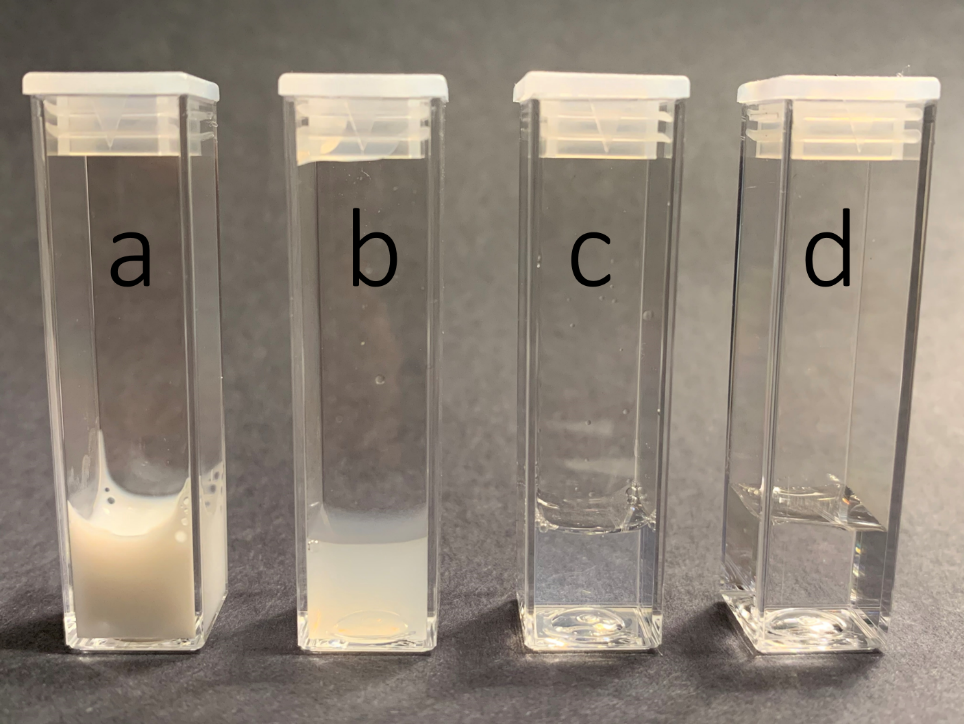


**Fig. S1** Standards that were used to demonstrate both multiple and single scattering include: a) stock 300nm standard, b) 300nm standard diluted 100-fold, c) stock 20nm standard, and d) water (for visual comparison).


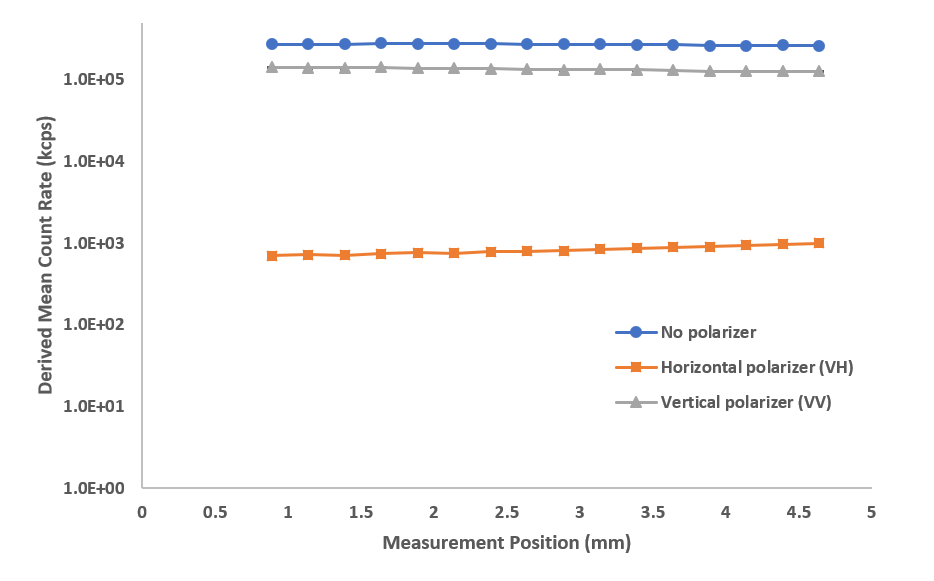


**Fig. S2** Derived count rate as a function of measurement position at different polarizer configurations for stock 20nm latex standards. The solution exhibits complete single scattering with minimal change in count rate over position and minimal cross-polarized signal (low signal in the horizontal plane).


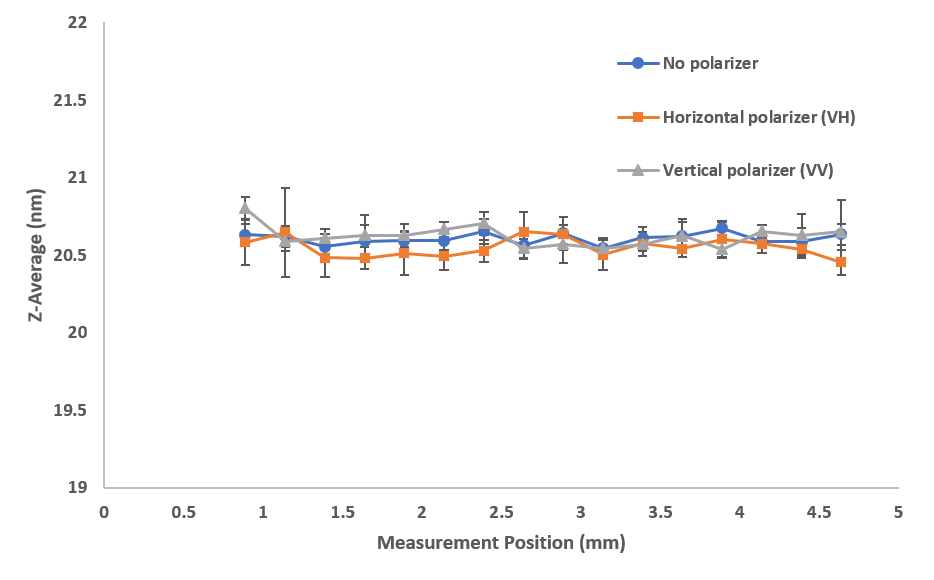


**Fig. S3** Size (z-average diameter) as a function of measurement position shows the absence of multiple scattering with different polarizer configurations for stock 20nm latex standards. The sample shows single scattering with consistent sizes over position regardless of polarizer configuration.


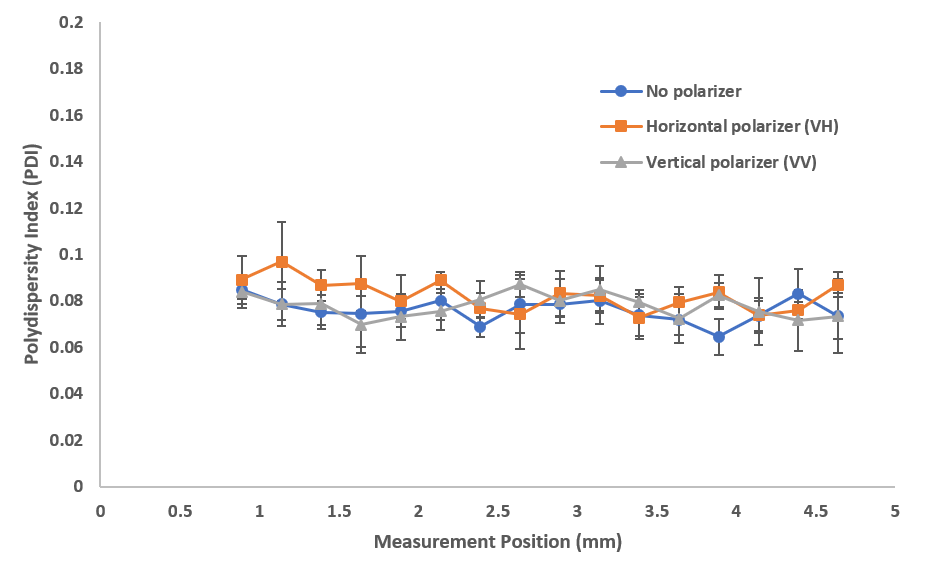


**Fig. S4** Polydispersity index (PDI) as a function of measurement position shows the absence of multiple scattering with different polarizer configurations for stock 20nm latex standards. Single scattering shows consistent and low PDIs regardless of polarizer configuration.


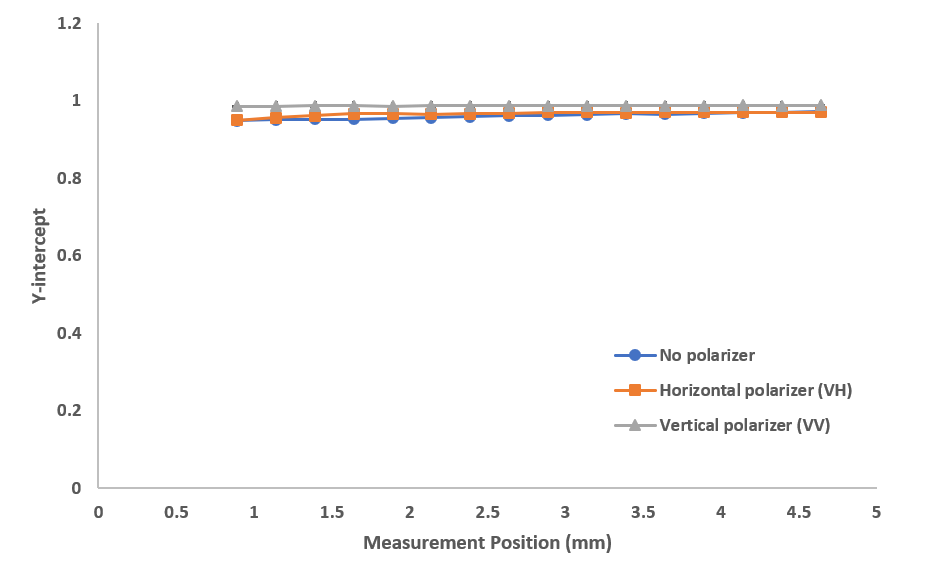


**Fig. S5** Y-intercept as a function of measurement position for stock 20nm latex standards showing reasonable scattering intensity, any polarizer detection will lead to a near-perfect intercept approaching 1.


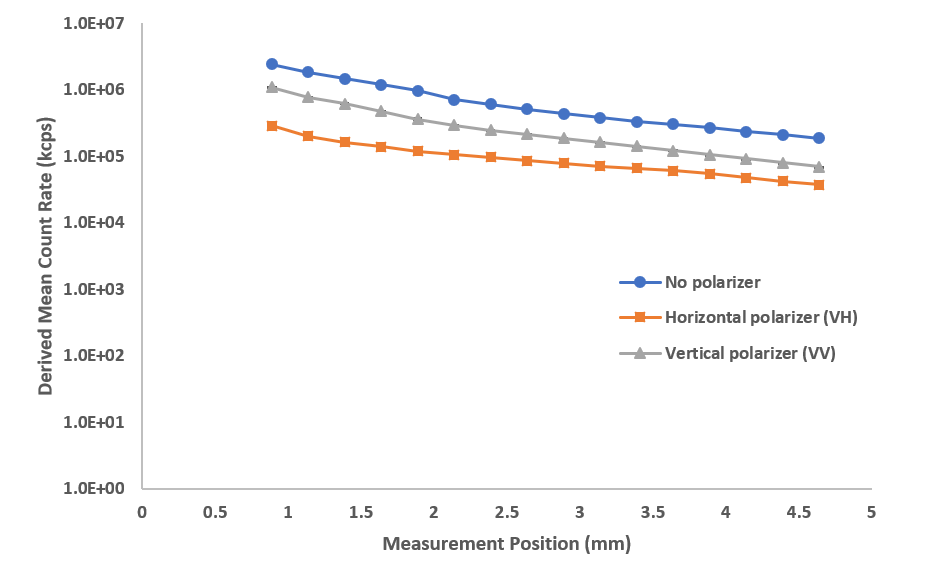


**Fig. S6** Derived count rate as a function of measurement position at different polarizer configurations for stock 300nm latex standards. The influence of multiple scattering increases further into the sample, away from the cuvette wall, leading to an apparent decrease in scattering intensity.


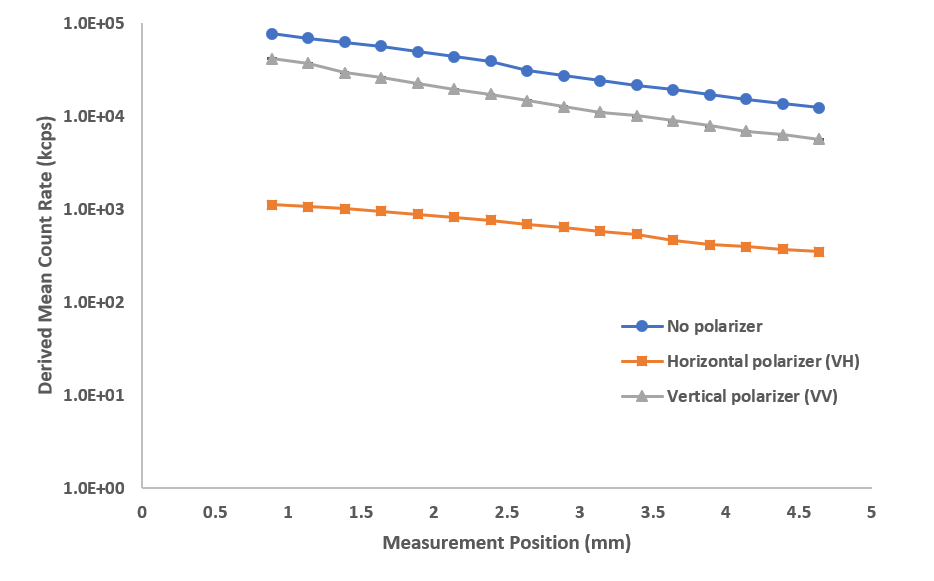


**Fig. S7** Derived count rate as a function of measurement position at different polarizer configurations for 100-fold diluted 300nm latex standards. The solution exhibits less multiple scattering with less change in count rate over position and minimal cross-polarized signal (low signal in the horizontal plane) when compared to the stock solution. The count rate scale shows orders of magnitude difference between stock and 100-fold diluted 300nm standards. Dilution in water without a stabilizing buffer might cause the 300nm latex standards to adhere to the cuvette thus exhibiting higher concentration at the cuvette wall.


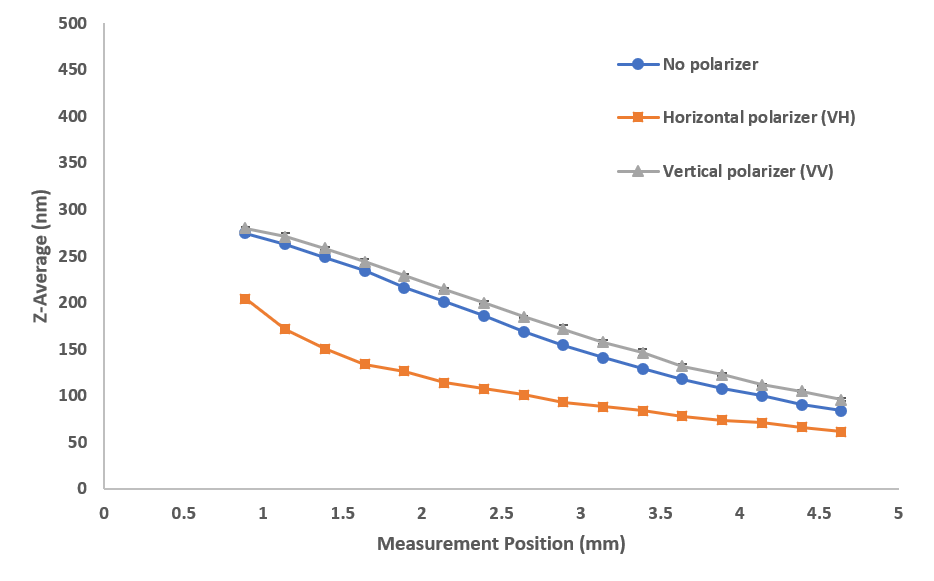


**Fig. S8** Size (z-average diameter) as a function of measurement position shows the effects of multiple scattering with different polarizer configurations for stock 300nm latex standards. The significance and influence of multiple scattering increases further into the sample, away from the cuvette wall.


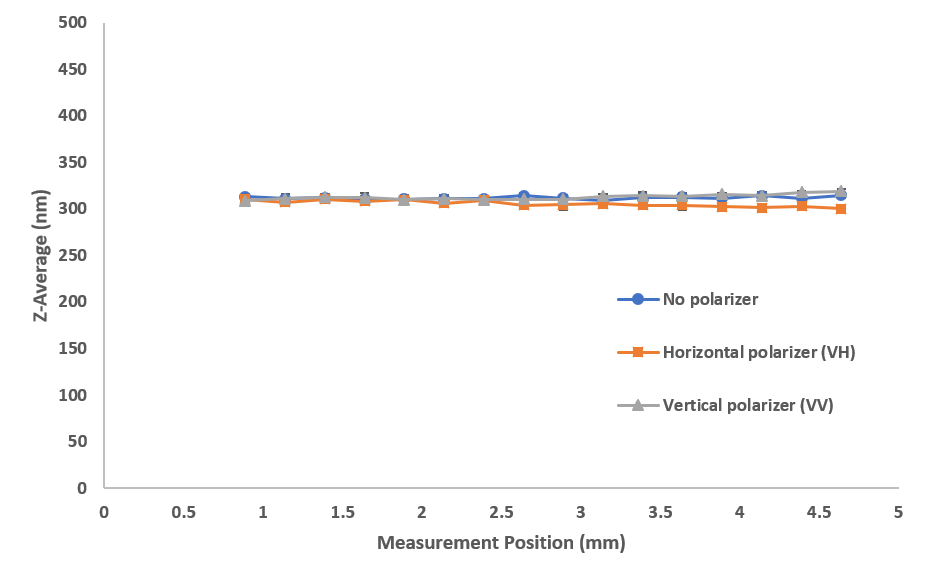


**Fig. S9** Size (z-average diameter) as a function of measurement position shows the absence of multiple scattering with different polarizer configurations for 100-fold diluted 300nm latex standard. The sample shows single scattering with consistent sizes over position regardless of polarizer configuration.


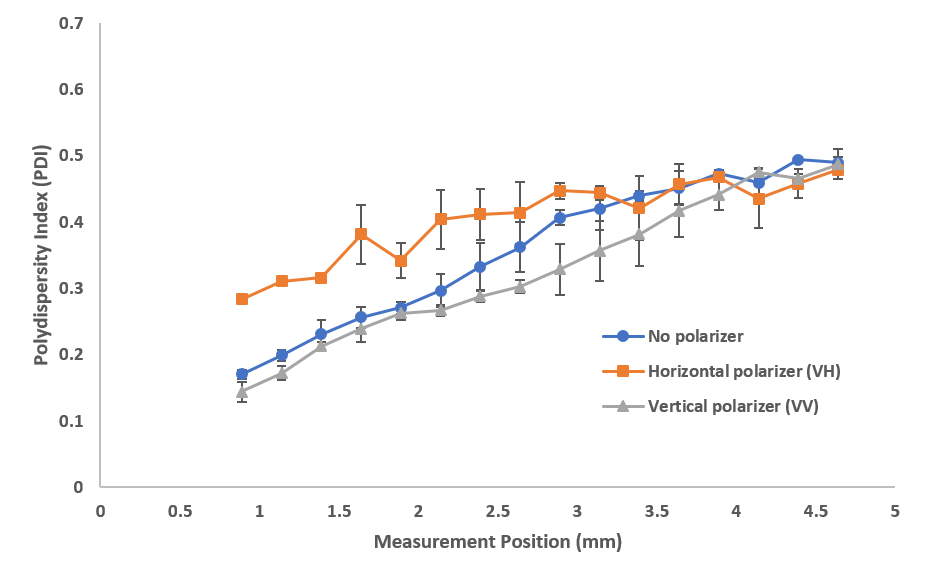


**Fig. S10** Polydispersity index (PDI) as a function of measurement position shows the effects of multiple scattering with different polarizer configurations for stock 300nm latex standards. The effects of multiple scattering are most evident at positions closer to the cell wall


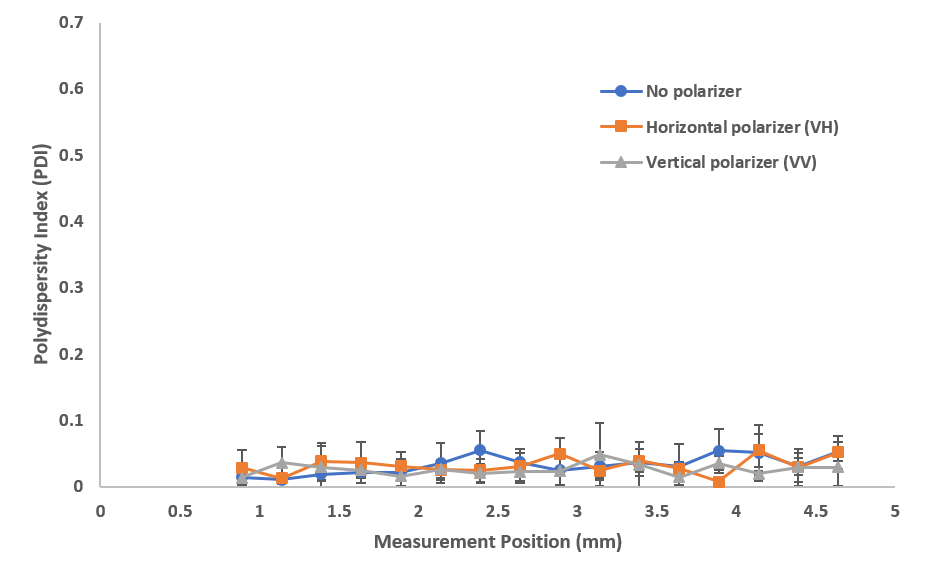


**Fig. S11** Polydispersity index (PDI) as a function of measurement position shows the absence of multiple scattering with different polarizer configurations for 100-fold diluted 300nm latex standards. Single scattering shows consistent and lower PDIs regardless of polarizer configuration.


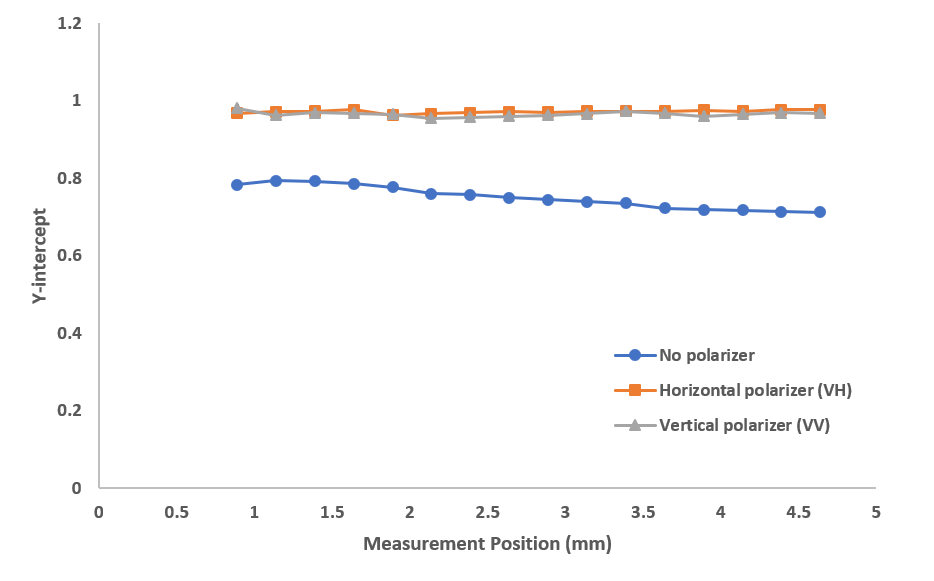


**Fig. S12** Y-intercept as a function of measurement position for stock 300nm latex standards showing increased presence of multiple scattering away from the cuvette wall. For reasonable scattering intensity, any polarizer detection will lead to a near-perfect intercept approaching 1.


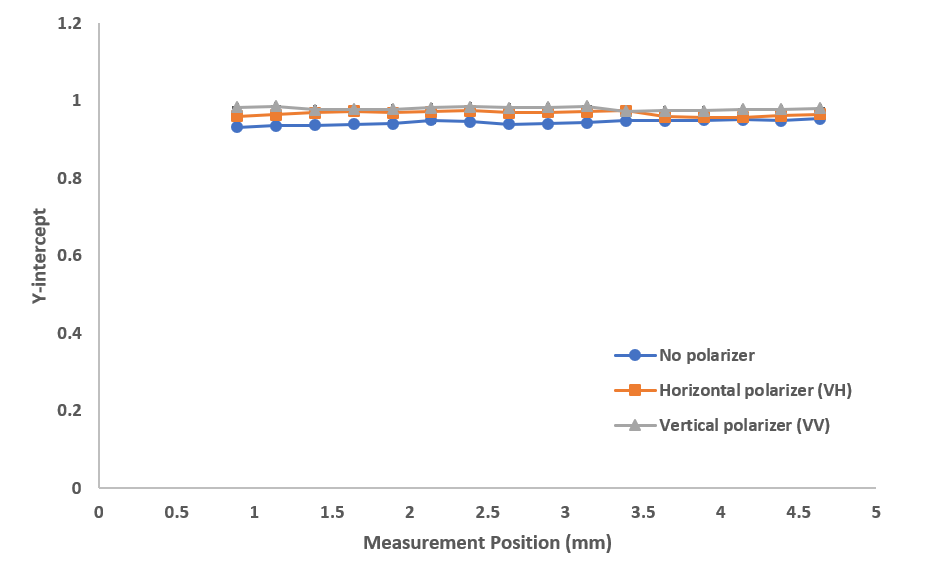


**Fig. S13** Y-intercept as a function of measurement position for 100-fold diluted 300nm latex standards showing reasonable scattering intensity, any polarizer detection will lead to a near-perfect intercept approaching 1.
